# Digital image processing realized by memristor-based technologies

**DOI:** 10.1186/s11671-023-03901-w

**Published:** 2023-09-28

**Authors:** Lei Wang, Qingyue Meng, Huihui Wang, Jiyuan Jiang, Xiang Wan, Xiaoyan Liu, Xiaojuan Lian, Zhikuang Cai

**Affiliations:** College of Integrated Circuit Science and Engineering, Nanjing University of Posts and Telecommunications, Nanjing, 210023 China

**Keywords:** Image processing, Memristor, Array-level networks, Neuromorphic system, Computer-in-memory

## Abstract

Today performance and operational efficiency of computer systems on digital image processing are exacerbated owing to the increased complexity of image processing. It is also difficult for image processors based on complementary metal–oxide–semiconductor (CMOS) transistors to continuously increase the integration density, causing by their underlying physical restriction and economic costs. However, such obstacles can be eliminated by non-volatile resistive memory technologies (known as memristors), arising from their compacted area, speed, power consumption high efficiency, and in-memory computing capability. This review begins with presenting the image processing methods based on pure algorithm and conventional CMOS-based digital image processing strategies. Subsequently, current issues faced by digital image processing and the strategies adopted for overcoming these issues, are discussed. The state-of-the-art memristor technologies and their challenges in digital image processing applications are also introduced, such as memristor-based image compression, memristor-based edge and line detections, and voice and image recognition using memristors. This review finally envisages the prospects for successful implementation of memristor devices in digital image processing.

## Introduction

Digital image processing technology, a technique for processing image information with computers or real-time hardware, mainly involves image coding and compression, image enhancement and restoration, image segmentation, image recognition, and so on. Common algorithms include the familiar single/multi-scale retinex algorithm for image enhancement [1, 2], Sobel [3] and Canny operators for image edge detection [4], and Gaussian filtering algorithm for image denoising [5]. Table 1 summarizes the classical algorithms used in image enhancement and restoration, image segmentation and recognition classification processing. Although computer image processing is highly sophisticated and abundant, the processing speed is not ideal for complex or real-time situations. Therefore, researchers are always keen to explore image processing schemes that conform to the human visual system inspired by the neuromorphic system.

**Table 1:** Summary of some classical algorithms and types of memristor used in digital image processing

Image processing	Classification		Types of memristor used
Enhancement and reconstruction			
Enhancement algorithm	Histogram equalization	Histogram Equalization algorithm (HE)	
		Adaptive Histogram Equalization algorithm (AHE)	
		Contrast Limited Adaptive Histogram Equalization (CLAHE)	
		Local Region Stretch (LRS) Histogram Equalization	
	UnSharp Masking (USM)		Ni/NiO/Ni [31]
	Retinex	Single Scale Retinex (SSR)	TiN/TEL (Thermal Enhanced Layer)/HfOx/TiN [32]
		Multi-Scale Retinex (MSR)	Ag/HfO_2_/Ag_2_S/Ag [33]
			Gale memristor model (HP) [34]
		Multi-Scale Retinex with Color Restoration (MSRCR)	Au/Ag–TiO_2_/FTO (Fluorine-doped Tin Oxide) [35]
Restoration algorithm	Frequency domain	Inverse Filtering	
		Wiener Filtering	
		Kalman Filtering (Recursive Filtering)	
	Regularized Filtering		
	Adaptive restoration algorithm		
	Lucy–Richardson		
Segmentation			
Threshold value-based	Peak_to_valley Method Based on Gray Histogram		
	OTSU		
	Minimum Error (ME)		
	Maximum Entropy Method (MEM)		
Edge-based	First order differential operator	Robert	
		Sobel	Gale memristor model (HP) [34]
		Prewitt	Pt/HfO_2_/Ta [36]
		Canny	Ti/PdSeO_x_/PdSe_2_/Au [37]
	Second order differential operator	Kirsh	
		Laplacian	
		Laplacian of Gaussian (LoG)	
Deep learning	Fully Convolutional Networks (FCN), U-net, SegNet, Mask R-CNN		
Classification and recognition			
Feature-based	Color features, geometric features, texture feature		Pt/TiNx/PCMO(Pr_0.7_Ca_0.3_MnO_3_)/Pt [38]
Deep Learning	LeNet, AlexNet, GoogLeNet		TiN/TaO_x_/HfO_x_/TiN [39]
			ITO/MXene-ZnO/Al [40]

Owing to the increasing demand for high-performance computing in the digital image processing field, traditional Von Neumann computer architectures become highly inefficient. In recent years, neuromorphic hardware systems have gained significant attention. Such systems can potentially provide bio-perception and information processing capabilities within a compact and energy-efficient platform [6, 7]. Inspired by the human brain, neuromorphic computing can address the inherent limitations of traditional von Neumann architectures [8]. Therefore, the construction of biorealistic synaptic primitives with rich spatiotemporal dynamics is indispensable for low-power neuromorphic hardware [9]. Neuromorphic vision has special advantages over conventional machine vision and has attracted considerable interest due to its ability to imitate human visual perception [10–12].

Traditional digital image processing architectures that incorporate neuromorphic systems have inherent parallel processing capabilities of cellular nonlinear network (CNN)-based architectures, making them effective platforms for various image processing tasks [13, 14], bringing scalability, simplicity, and power efficiency to VLSI implementations [15]. Sufficient CNN templates have been found to perform detail extraction tasks such as edge detection in vision systems [16]. Moreover, Johnson et al. [17–20] developed a pulse-coupled neural network (PCNN) by studying the γ-band synchronous spike dynamics. The concept of "pulse-coupled neural network" first appeared in [17] and the classical PCNN appeared in [21–23]. Pulse-coupled neural networks simulate the behavior of optic nerve cells in the visual cortex of mammals (e.g., cats) [18, 19, 24]. It was shown that PCNNs are single-layer neural networks with a two-dimensional matrix structure, whose size and layout depend on the input image. It is promising for real-time image processing as it can perform image fusion [25, 26], image segmentation [27, 28], and object detection [29, 30] without requiring training process.

However, the architectures mentioned above involve complex software algorithms, which intangibly add processing time and reduce the system efficiency. Therefore, researchers are inspired by the human visual system and try to establish a novel digital image processing architecture. The human visual system (HVS) integrates perceptual and processing, which involves filtering or suppressing noise and enhancing target features with the retina, followed by parallel high-level image processing in the visual cortex. [11, 12, 41–43]. In digital general-purpose processors, many image processing applications require multiple operations per second, even though these applications do not require floating-point precision [44]. In a memristor-based image processing network, the image processing time and iterations required for the program are directly reduced on account of the fast-switching speed and low power consumption of the memristor, which can not only store information but also compute and process it.

Algorithms with memristor behavior have an impact on digital image processing as they introduce nonlinear effects in digital image processing algorithms that may lead to more complex and diverse image processing results. In addition, some complicated algorithms require a considerable computational resource, resulting in slower operation and the need to optimize the algorithm or use better computer hardware. Therefore, it is necessary to carefully consider the effects of the memristor behaviors on the processing results when applying them to digital image processing algorithms and to select appropriate processing methods to control and adjust their effects to meet specific image processing demands. In terms of digital image processing results, the quality of images processed in this way is not optimal when dealing with images generated under special conditions (e.g., poor lighting conditions, excessive noise, etc.) or large-scale image data. Therefore, to reduce power consumption and training costs, hardware digital image processing architectures based on memristor networks that enable massively parallelism and minimize data transfers have emerged.

**Fig. 1:**
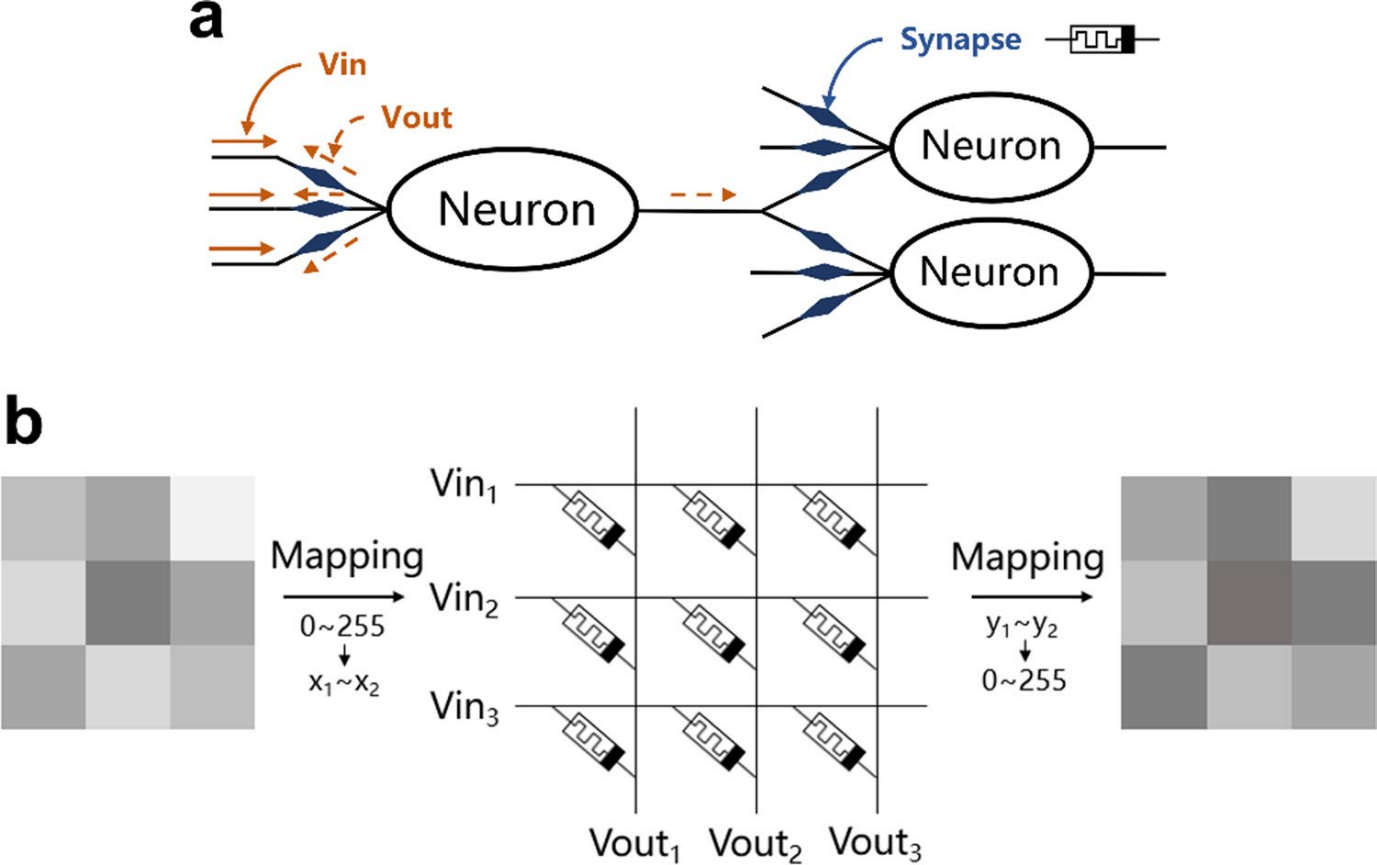
Digital image processing architecture for neuromorphic systems combined with memristors. **a** Diagram of the interaction between biological neurons. **b** Process of 3 × 3 Gy image processing based on memristor array, take enhancement as an example

Memristor is the crucial component for the analog visual system's enhancement and inhibition effects. The principle of lateral inhibition of biological neurons is shown in Fig. 1a. When a neuron is excited through stimulation, and a neighboring neuron is stimulated, the excitation occurring in the neighboring neuron has an inhibitory effect on the former, and this feature coincides with the properties of the memristor. The memristor, introduced by L. Chua in 1971 [45] from the completeness of the circuit, is the fourth elementary two-terminal circuit element characterized by a nonlinear constitutive relationship between flux and charge and was consciously discovered by Strukov D B and his team in 2008 on the nanoscale metal oxides [46]. Moreover, memristors have optimal write energy and standby power, where the majority of pulse-code modulated (PCM) devices and resistive random access memories (RRAM) have write energies of about 10–100 pJ and 100 fJ–10 pJ [47], respectively. Several studies have proved that the computational energy efficiency of memristors exceeds that of today's graphics processing units by two orders of magnitude [48]. Here, the enhancement process of a 3 × 3 grayscale image is used to explain the process of employing a memristor array structure to a hardware digital image, as shown in Fig. 1b. A two-dimensional image consists of many pixel points, and in view of a grayscale image, the grayscale value of the image is mapped to the input voltage (current) of the array, and the output voltage (current) is obtained through vector operation or interaction (enhancement or suppression) between the memristors by the array of equal size, and then the opposite step is performed as the previous one, i.e., the voltage or current is mapped to the grayscale values of 0 to 255. Finally, the processed results can be obtained. In memristor-based image processing networks, the fast-switching speed, and low power consumption of memristors directly reduce the image processing time and the iterations required for the program because of their capabilities not only for storing information but also for calculating and manipulating it.

In this article, we focus on helping the reader understand the current status of memristor devices and image processing based on memristor circuits. We present recent research on the application of memristors in hardware image processing and compare the implementation of pure software image processing and memristor-based image processing. Their advantages, disadvantages, and existing problems are subsequently analyzed. This paper is divided into four parts. “Memristor” section escribes the theory of memristors and presents the reasons for their application in the field of neuromorphology. “Image quality assessment metrics” section introduces several commonly used image evaluation metrics to facilitate later comparisons of the effects of memristor-based hardware digital image processing. “Discussion” section lists the current research on the application of memristor-based circuits in various aspects of image processing. Finally, we conclude with a discussion of the prospects for the development and openness of hardware digital image processing and summarize the work of this paper.

## Memristor

With the continuous development of big data, the Internet of Things, artificial intelligence and other technologies, it is urgent to put forward a new computing system to deal with dense data. The human brain can process and store data simultaneously, thus reducing energy consumption and greatly improving the efficiency of computing. Therefore, building brain-like operations and developing intelligent brain-like devices is an essential breakthrough in AI research [49]. Researchers at HP Labs have experimentally confirmed that memristors are a new type of nonlinear two-terminal nanoscale component with switching characteristics, memory capability, and continuous input and output properties [46]. Due to its inherent property of analog inputs and outputs, memristor-based memories can allow for higher accuracy than conventional binary memories. Compared to dynamic random access memory, memristors maintain their state after power loss, making memristor-based memories non-volatile [50, 51]. Notably, the combination of memristors and nanowire crossbar interconnection has become a topic of great interest to researchers [52, 53]. The memristor crossbar array structure combines the features of high storage density, high precision and fast access speed of memristors with the massively parallel processing of crossbar arrays, enabling the structure to possess strong information processing capabilities and easy compatibility with large-scale integrated circuits (VLSI). Considering the advantages above, it has broad application prospects in arithmetic operation, mode comparison, information processing, and virtual reality. This section introduces the memristors commonly applied in the hardware architecture of digital image processing and their working mechanisms. At the end of this section, we also summarize the electrical performance of memristors with different structures at the current stage (Table 2).

**Table 2:** Summary of electrical performance of memristors with different structures

Structure	Vset (V)	Vreset (V)	Switching ratio	Endurance (cycles)	Retention	Operation speed	RS mechanism	References
Pt/Ta_2_O_5−x_/TaO_2−x_/Pt	− 1	2	> 10	10^12^	–	> 10 ns	Schottky barrier	[73]
TiN/Ta_2_O_5_/TiN	2	− 2	360	–	–	< 10 ns	Conductive filament	[74]
Pt/SiOx:Pt/Ta	~ − 0.6	1 ~ 1.2	10^3^	3 × 10^7^	10^7^ s	< 100 ps	Ionic motion	[75]
ITO/LaAlO_3_/SrTiO_3_	− 1.3 ~ − 1.6	5	200	> 2000	10^4^ s	–	Conductive filament	[76]
Pt/TiN/PCMO/Pt	− 2.5	2	≈60	–	–	–	Schottky barrier	[77, 78]
Ta/HfO_2_/Pd	1.1	1.3	500	1.2 × 10^11^	≥ 10 years	≤ 5 ns	Conductive filament	[59]
Ag/N-GST/Pt	0.2	− 0.14	≈400	10^5^@85℃	–	–	Phase-change	[79]
ITO/Ag/MAPbI_3_/Au	2.4	− 2.2	≈10^7^	6 × 10^6^	4.2 × 10^7^ s	100 ps	Conductive filament	[80]
Au/MoS_2_/Au	3	− 2	10^7^	20	10^4^ s	–	Schottky barrier	[81]
Ag/FK-800/Pt	–	–	10^6^	106	> 10^6^ s	340us	Conductive filament	[82]
Au/Ag@MXene/TiO2/ITO	0.5	− 1.8	100	–	> 10^4^ s	< 300us	Schottky barrier	[83]
ITO/PVA-GO/ITO	0.2	− 0.2	> 10	500	10^4^ s	–	Conductive filament	[84]
Ag/(PEA)_2_Cs_3_Pb_4_I_13_/Pt	0.18	–	10^9^	200	2 × 10^3^ s	–	Schottky barrier	[85]
Au/SAM-SURMOF/Au	± 1	–	–	–	10^3^ s	–	Charge trapping/detrapping	[86]

### Memristors for image processing

**Fig. 2:**
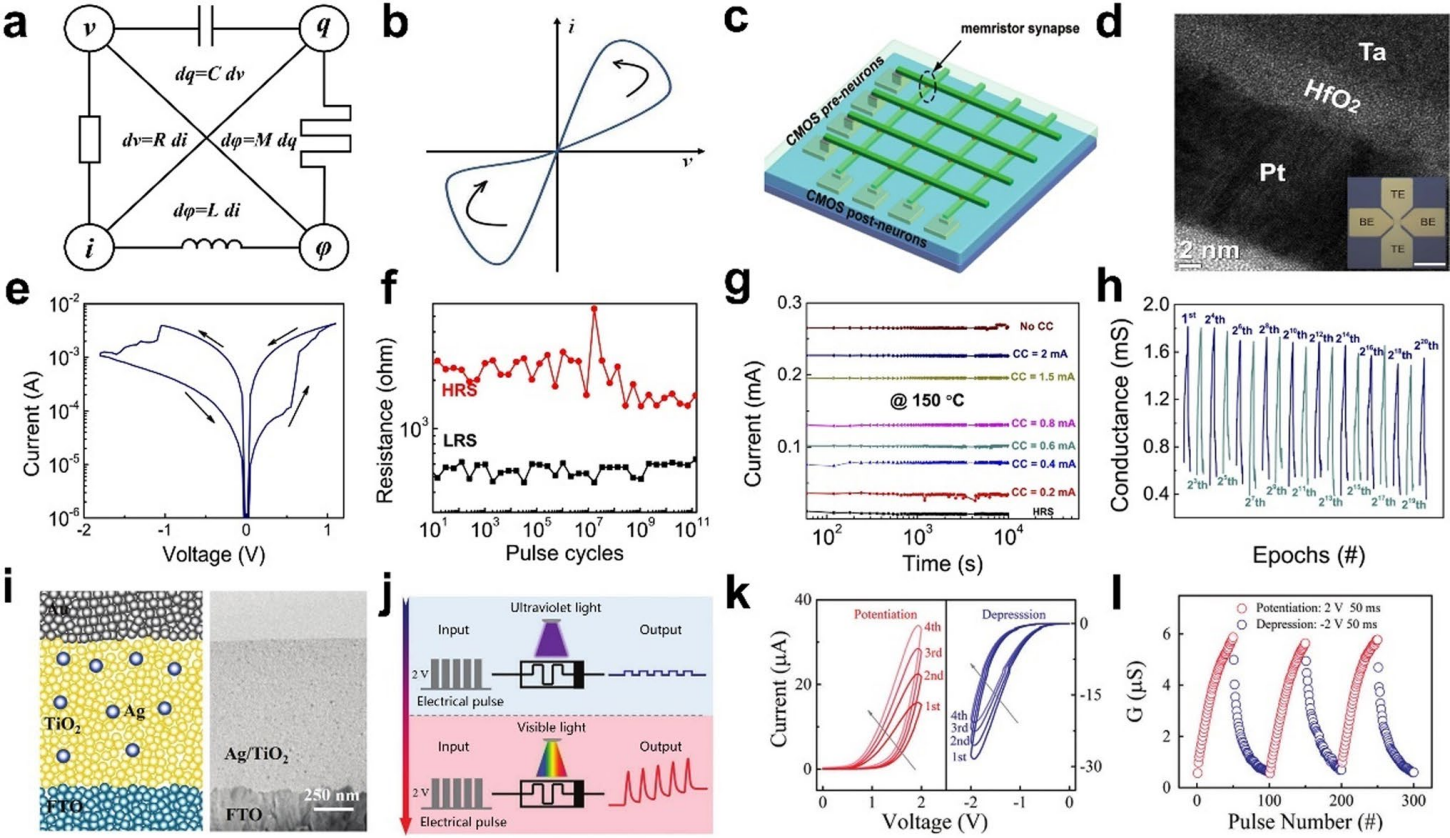
**a** Four basic circuit elements and their respective relationships. **b** A typical hysteresis loop of the memristor. **c** Diagram illustrating the structure of a neuromorphic crossbar comprised of memristor synapses and CMOS neurons [58]. **d** TEM cross-section of the Ta/HfO2/Pt device. Measurements run with the top Ta electrode biased and the bottom Pt electrode grounded [59]. **e** Typical I–V curve showing resistor switching behavior, with black arrows indicating device switching direction [59]. **f** High- and low-resistance states have been demonstrated for devices with 120 billion switching cycles at -3.05 V/100 ns RESET and 1.3 V/100 ns SET pulses [59]. **g** Retention testing of eight different levels at 150 °C (> 104 s) confirmed the non-volatile characteristics and demonstrated the device's suitability for multi-level memory [59]. **h** 2.^20^ enhancement/inhibition epochs were realized, each of these pulses comprising 39 pulses [59]. **i** Device structure and cross sectional TEM image of the Ag–TiO_2_ nanocomposite-based memristor [35]. **j** Schematic of optically gated electrically driven synaptic modulation operation [35]. **k** I–V curve of a memristor device after 15 min of exposure to visible light [35]. **l** Long-term conductance augmentation and inhibition stimulated by 50 positive/negative pulses (± 2 V, 50 ms) [35]

Memristor is a nonlinear resistor with memory capability whose resistance is affected by the amount of charge or magnetic flux passing through it. In 1971, Chua [45, 54] theoretically proposed the memristor (short for memory resistor) based on the symmetry argument of circuit theory. Memristance (resistance of a memristor) was defined as the ratio between the magnetic flux *φ* and charge *q* passing through the memristor (i.e., $M = {\text{d}}\varphi /{\text{d}}q$) by Chua (Fig. 2a). As *φ* and *q* are time integrals of voltage and current, respectively. Then,

1 $$M = \frac{{{\text{d}}\varphi /{\text{d}}t}}{{{\text{d}}q/{\text{d}}t}} = \frac{V}{I}$$

This equation shows that the unit of *M* is the same as the resistance, i.e., ohm (Ω). In 1976, Chua and Kang elucidated the strong dependence of memristive systems on the implementation of state variables and provided a generalized definition of memristive systems derived from memristors [54], which can be mathematically defined as:

2 $$v = R\left( {w,i} \right)i,$$

3 $$\frac{{{\text{d}}w}}{{{\text{d}}t}} = f\left( {w,t} \right),$$

where *w* is an internal state variable, and in general *R* and *f* are explicit functions of time. If an arbitrary periodic voltage (current) signal is applied to an ideal memristor and the excitation voltage (current) and response voltage (current) are then plotted, a diagonal "8"-shaped tight pinch hysteresis return is obtained, as shown in Fig. 2b, which was used by Chua as a landmark criterion for memristor phenomena [55]. This definition was eventually refined in Chua's latest publication [56, 57]. This pinched hysteresis loop of current voltage (*i* − *v*) has also become the most representative feature of the memristor. The shape of this loop varies with the amplitude and frequency of the input waveform, but the common feature is the absence of positive and negative values in each cycle and the passage through the origin of the coordinates. Meanwhile, direct experimental support for memristor neuromorphic systems such as spike-timing-dependent plasticity originated from a hybrid system of memristor synapses and CMOS neurons (Fig. 2c).

Here we focus on two typical types of memristor structures and device performance applied in digital image processing. Jiang et al. reported a Ta/HfO_2_/Pt memristor (Fig. 2d) [59] with low programming voltages (Fig. 2e), fast switching speeds (≤ 5 ns), high endurance (120 billion cycles) (Fig. 2f) and reliable retention (> > 10 years extrapolated at 85 °C). In addition, potentiation and depression were demonstrated over 2^20^ epochs (Fig. 2h), indicating that the device can be used for multi-level non-volatile memories (Fig. 2g) and neuromorphic computing applications. Shan et al. developed a plasmonic optoelectronic memristor [35] (Fig. 2i) that relies on optical excitation in an Ag-TiO_2_ nanocomposite film and the effects of localized surface plasmon resonance (LSPR). Fully light-induced and light-gated synaptic plasticity functions were achieved in the single device (Fig. 2j), including reversible synaptic potentiation/suppression under visible and ultraviolet illumination and modulation of the STDP learning rule (Fig. 2k, l), which can be utilized for visual sensing and low-level image pre-processing (including contrast enhancement and noise reduction).

### Working mechanism

**Fig. 3:**
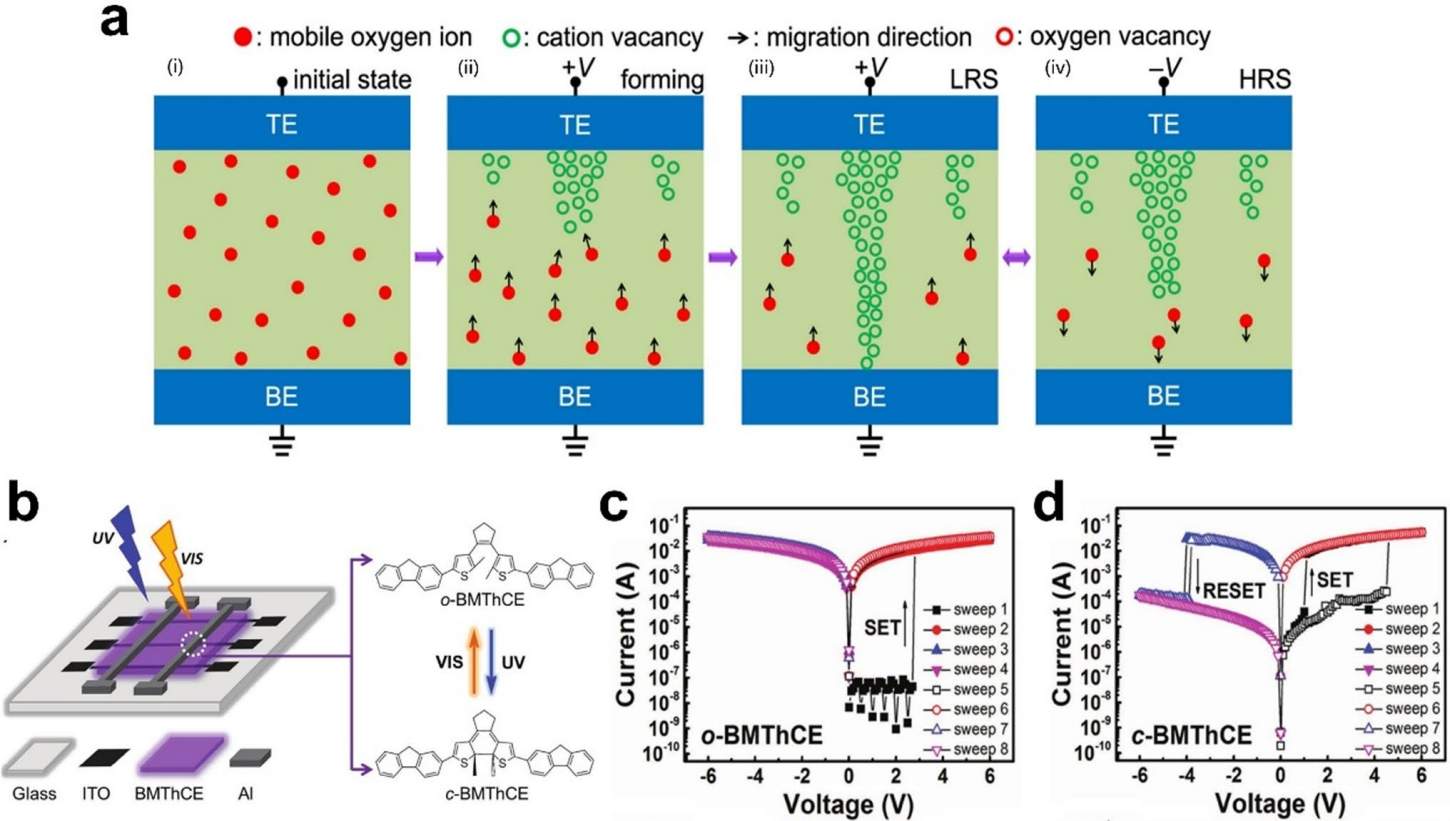
**a** Schematic of anion migration dominated switching kinetics in p-type semiconductors. (i) The initial state with random distribution of mobile oxygen ions. (ii) The nucleation and subsequent growth of p-type CFs composed of cation vacancies from anode to cathode during the forming process. (iii) Full CF LRS in the thinnest region near the cathode. (iv) The thinnest region of the CF portion ruptured by the HRS [65]. **b** Schematic of BMThCE-based device, and the chemical structures of the photochromic diarylethene (UV: ultraviolet light; VIS: visible light) [35]. **c** I–V characteristics of the BMThCE-based memories ITO/o-BMThCE/Al and **d** ITO/c-BMThCE/Al [35]

The mechanism lies in the fact that synapses are intrinsically two-terminal devices, which share a striking similarity with memristive devices [45, 46]. The advantage of this structure is that it can potentially provide connectivity and functional density comparable to biological systems, rather than operating in a digital computer manner [60]. These devices consist of a simple metal − insulator − metal (MIM) layer structure. The forming process creates localized conducting filaments, and the movement of these filaments leads to discrete and abrupt resistive switching characteristics [51, 61–64]. Specifically, the switching kinetics dominated by anion migration in semiconductors can be understood as follows. There are some mobile oxygen ions in the p-type storage medium, as schematically illustrated in Fig. 3a-i. These moving oxygen ions migrate toward the TE when the top electrode (TE) is positively biased and then accumulate near the TE, thus creating a large number of cationic vacancies in the TE (Fig. 3a-ii). Once the fully p-type semiconductor conducting filament is formed, the device will switch to the low-resistance state (LRS) (Fig. 3a-iii). Most of the Joule heat will be generated at the thinnest part of the conducting filament when TE is negatively biased, greatly accelerating the movement of oxygen ions in that region. The oxygen ions flowing in this region will rapidly migrate toward the BE driven by the electric field, and as a result, the concentration of cationic vacancies at the thinnest part of the CF of the p-type semiconductor will be significantly reduced, resulting in the CF breaking off there, at which point the device is in the high-resistance state (HRS) (Fig. 3a-iv). When semiconductor (TiOx) junctions/two dynamic metal (Pt) are operated in series, a range of device states occur.

Slightly different from electrically induced RS memories, the physical mechanisms of optical effects in optical memristors include photovoltaic effects and light-induced chemical reactions/configuration changes, etc. Photovoltaic effects typically involve the creation of free carriers, the separation of photogenerated electron–hole pairs, and the generation of voltages or currents from incident photons [66]. The separation of electron–hole is highly correlated with the Schottky barrier between metal and semiconductor or the internal electric field induced by the heterojunction interface (heterojunction system) [67]. This causes the holes to move toward positive electrode and electrons to the negative electrode, which subsequently extracts charge to the external circuit and generates an open circuit voltage. Photochemical reactions entail photons absorption, which excite molecules and cause chemical changes such as ionization and isomerization [68] (Fig. 3b). The photo-induced switching behavior is tightly linked to conformational changes within the photoactive material, which may lead to changes in chemical bonds and energy bands. The photo-induced transition between conformational structures does have a remarkable impact on the RS type as the energy level changes, which can greatly modulate the device performance in a precise and energy-efficient manner (Fig. 3c,d).

Reportedly, memristors respond to light and electrical stimuli [69–72]. Neuromorphic computing implementations in the electrical and optical domains requires a full combination of the integrated processing power of the electrical domain and low energy consumption and high bandwidth of the optical fields. Memristors have become both state modulators and photodetectors for their particular characteristics, capable of processing both electrical and optical signals. The common methods to realize synaptic or neuronal behavior include modulating the memristor state with electrical and optical programming signals, i.e., resistance or optical transmittance. In addition, the programmed input and readout signals are located in different domains, thus enabling direct conversion of optoelectronic signals, which is extremely attractive. For example, an electrical (optical) signal can change the optical (electrical) signal in a state modulator (photodetector).

## Image quality assessment metrics

Image quality assessment metrics play an important role in various image processing applications. Digital images suffer from various distortions during the process of acquisition, processing, compression, storage, transmission and reproduction, any of which may leads to a degradation of visual quality. Image quality assessment metrics are available for optimizing algorithms and parameter settings of image processing systems and benchmarking them, and dynamically monitoring and adjusting image quality. Two types of metrics exist for assessing image quality, subjective and objective image quality assessment metrics. They are briefly described below.

### Subjective image quality assessment metrics

Subjective assessment, also called subjective evaluation, is to evaluate the quality of an image through the subjective perception of a person as an observer and can most truly reflect the human visual perception. Common subjective evaluations are absolute and relative evaluations. The former involves the observers rating the original image and the image to be evaluated, and the latter involves the observers comparing the given image based on their own subjective feeling without any reference. The final evaluation score for both methods is the average of each evaluation score.

The subjective evaluation criterion uses the Mean Opinion Score (MOS):

4 $${\text{MOS}} = \frac{{\mathop \sum \nolimits_{i = 1}^{k} N_{i} S_{i} }}{{\mathop \sum \nolimits_{i = 1}^{k} N_{i} }}$$

where $k \in \left\{ {1,2, \ldots K} \right\}$ is the evaluation level of the observer, *S*_*i*_ is the evaluation score corresponding to the level, and *N*_*i*_ is the number of evaluators for each type of score.

### Objective image quality assessment metrics

Unlike the subjective assessment of images, objective evaluation assesses the quality of the image by establishing a mathematical model, scoring the image texture, sharpness, focus and other aspects and calculating the results, which can scientifically reflect the human eye's subjective perception of the image. It can be divided into full-reference, half-reference and no-reference image quality assessment methods according to whether the corresponding reference image can be found [87]. This section presents several common objective image quality assessment metrics, which are as follows.

Mean Square Error (MSE): an expected value of the squared difference between the true and estimated values of a parameter. Assuming that the reference image is *f*, image to be measured is *g*, and size of two images is *M* × *N*. The grayscale values of the pixels are noted as *f*(*i*, *j*), *g*(*i*, *j*), and the mean squared error can be expressed as:

5 $${\text{MSE}} = \frac{1}{M \times N}\mathop \sum \limits_{i = 1}^{M} \mathop \sum \limits_{j = 1}^{N} \left[ {f\left( {i,j} \right) - g\left( {i,j} \right)} \right]^{2}$$

Peak Signal to Noise Ratio (PSNR): a calculation of the ratio of the maximum power of a signal to the power value of the noise. The larger the value, the smaller the distortion. The formula for calculating the PSNR is shown in Eq. (6).

6 $${\text{PSNR}} = 10\log_{10} \frac{{255^{2} }}{{{\text{MSE}}\left( {f,g} \right)}}$$

Structural Similarity (SSIM): A well-known qualify metric developed by Wang et al. [87] for measuring the similarity between two images. It is thought to be associated with the perception quality of the HVS. SSIM is designed to model any image distortion as a mixture of three factors, loss of correlation, contrast distortion, luminance distortion. The SSIM is defined as:

7 $${\text{SSIM}}\left( {f,g} \right) = l\left( {f,g} \right)c\left( {f,g} \right)s\left( {f,g} \right)$$

where

8 $$\left\{ {\begin{array}{*{20}l} {l\left( {f,g} \right) = \frac{{2\mu_{f} \mu_{g} + C_{1} }}{{\mu_{f}^{2} + \mu_{g}^{2} + C_{1} }}} \hfill \\ {c\left( {f,g} \right) = \frac{{2\sigma_{f} \sigma_{g} + C_{2} }}{{\sigma_{f}^{2} + \sigma_{g}^{2} + C_{2} }}} \hfill \\ {s\left( {f,g} \right) = \frac{{\sigma_{fg} + C_{3} }}{{\sigma_{f} \sigma_{g} + C_{3} }}} \hfill \\ \end{array} } \right.$$

Note that C1, C2, C3 are positive constants, aiming at avoiding the denominator to be 0 and *σ*_*fg*_ is the covariance between *f* and *g*. The first item in (8) is the luminance comparison function which indicates the proximity of the average brightness of two images (*μ*_*f*_ and *μg*). This factor acquires the maximum 1 only if *μ*_*f*_ = *μ*_*g*_. The second one is the contrast function, which measures how closely two images compare, where contrast is measured in terms of standard deviation *σ*_*f*_ and *σ*_*g*_. The maximum value of this term is 1 only when *σ*_*f*_ = *σ*_*g*_. The last one is the structural contrast function representing the relevant coefficient between the two images *f* and *g*. Hence, the positive value range of SSIM is [0, 1], where the value of 1 means that *f* = *g* and 0 means no correlation between images.

## Applications of memristor in digital image processing

Memristors have been widely employed in simulating artificial synapses because of their complex analog behavior since the rediscovery of the reversible resistive switching effect. Meanwhile, memristors can also be integrated with CMOS logic devices to serve as programmable switches [88], logic units [89, 90], etc. With the development of CMOS technology, it has allowed the large-scale integration of integrated and excited (I&F) neurons on a single chip [91–94]. To overcome the need for CMOS circuits with numerous transistors and high-power consumption, it was found that memristor (memory + resistor) devices were invented and successfully used as synapses with low power consumption and high speed [95–98]. The computing and storage capabilities exhibited by the memristor crossbar array are expected to relieve the Von Neumann bottleneck, save the required area and energy, and increase the computing speed, which can be used in the fields of image processing and neural networks.

### Image logical operation

Image logic operations, also known as image Boolean operations, are implemented based on pixels between two or more images [99], and the grayscale distribution of the operation results is different from that of the participating operations, mainly including AND, OR, NOT and XOR. In the field of image processing, AND and OR operations usually act as templates to extract sub-images from an image. NOT operation is used to reverse an image, thereby enhancing it. XOR operation is applied to encrypt and decrypt an image. They are commonly employed in the pre-processing of complex image processing, such as image segmentation, target detection and recognition, etc.

A class of advanced vision microprocessors was previously integrated on the same small hardware platform based on cellular neural networks (CNNs) [100], general-purpose computers [101], and CMOS light detection matrices. Photodetector-acquired data in these intelligent sensor arrays were processed under a set of local and global rules that were applied simultaneously and equally to each cell's neighborhood, endowing CNN’s general-purpose machines with massively parallel computing capabilities. The spatial resolution of these processors is rather low due to the limitation of the processing units that can be integrated within the integrated circuit area although these advanced processors processed images at a relatively fast speed.

**Fig.4:**
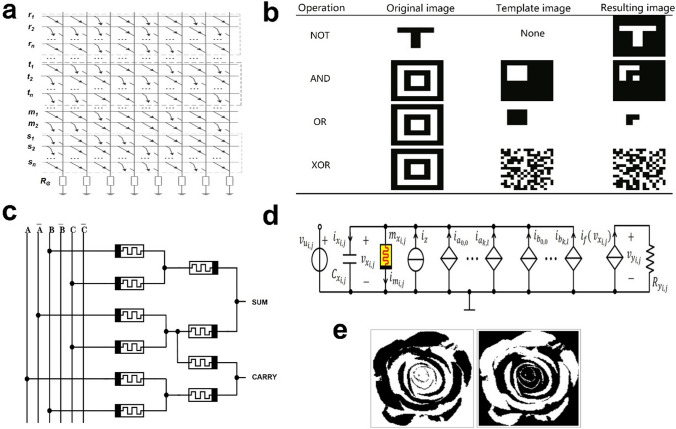
**a** Resulting image of the XOR operation stored in memristor memory [102]. **b** Images used in logic operations [102]. **c** Schematic of proposed memristor-based one-bit approximate full adder [104]. **d** Circuit implementation of the M-CNN processing element C (i, j) (i ∈ {1, … M}, j ∈ {1, … N}). The capacitor, memristor, and resistor are unvaried from cell to cell, i.e., Cxi, j = Cx, mxi, j = mx, and Ryi, j = Ry [105]. **e** Input binary image and output binary image visualizing the data stored in the memristances at steady state [105]

To address the above problems, Zhou et al. [102] gave a memristor-based architecture combining memory and image processing functionalities. Unlike conventional memory systems, the architecture can perform image logic operations with the assistance of extra memristors, where the system is composed of a memory array, computing array, simulating voltage generator, address system, and a read and writing system. They considered the NOT, AND, OR, and XOR operations on images. In Fig. 4a, b, the experimental results showed that the architecture was functionally correct and could reduce memory access considerably compared to the scheme proposed in [103]. However, this architecture has a problem that the running memristor needs to be reset when the next image processing operation occurs. How to correctly select the operating memristor will be the problem to be solved.

Subsequently, Muthulakshmi et al. proposed a plausible approach [104] toward approximate computing with memristors for designing an 8-bit Ripple Carry Adder (RCA), which performed bitwise pixel addition of two grayscale images with the same size and compared the design with images obtained by the exact addition method. The researchers found that the delay of one-bit accurate and approximate adder was obtained as 32.56 and 0.2316 ns, respectively, demonstrating a significant reduction in the delay. Figure 4c briefly draws a one-bit approximate full adder based on the memristor. As far as Lena's image processing results are concerned, parameters values like Structural Content (SC), MSE, PSNR, Mean Absolute Error (MAE), and Normalized Absolute Error (NAE) were found low, as all bits were approximated by the worst-case scenario, in contrast to the findings of Almurib et al. [106].

In recent years, Ronald Tetzlaff et al. proposed a new memristive computing paradigm theory based on CNNs [105], which laid a theoretical foundation for the realization of the new memristive computing paradigm of Memristor-CNNs(M-CNNs). In this theory, inserting non-volatile memristors, which endowed dynamic arrays with the ability to store data into and retrieve data from resistively switched memristors, reduces the use of additional memory modules. Figure 4d shows the circuit diagram of the M-CNNs unit. The left side of the circuit is an independent power supply, and the original linear resistance in the circuit is replaced by a non-volatile memristor. Figure 4e is the binary raw input image and the encoded image stored in the memristor, which is the complement of the original image. Images can be encoded in the memristor cellular array, which opens a new direction for future image processing. Nevertheless, the proposed framework relies on the combination of memristor and logic operation algorithms more than the system program of the entire memristor networks, and it is not generalized for the processing of digital images. However, it still provides a referable idea for memristor-based hardware digital image processing.

### Image compression

**Fig. 5:**
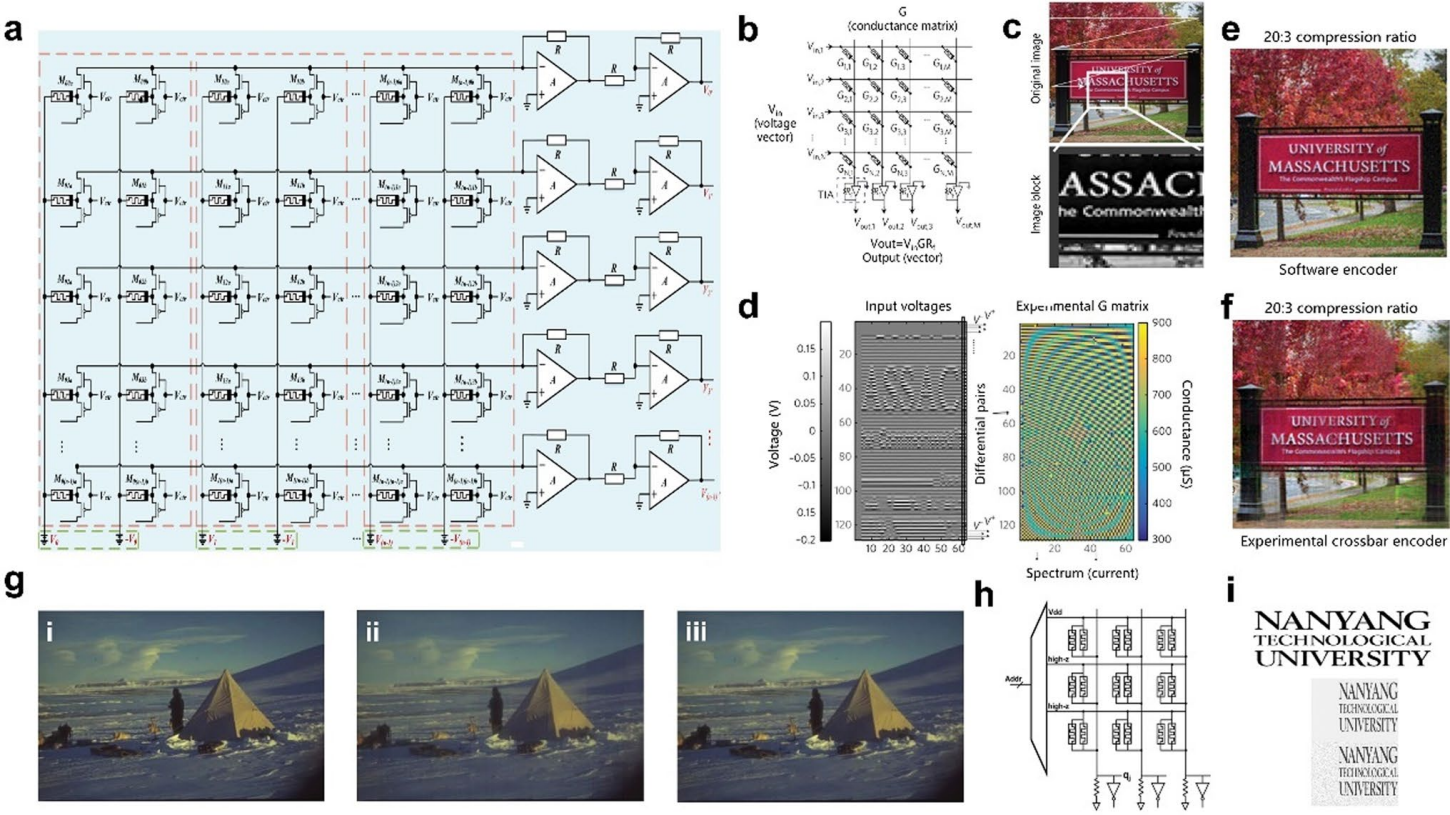
**a** Circuit Design of DCT and IDCT [107]. **b** Schematic diagram of vector–matrix multiplication operation (VMM). Multiplication is performed by Ohm's law, where the injection current is the product of the voltage applied across the row and the conductance of the crossing cells, and the currents on each column are summed according to Kirchhoff's current law. The total current from each column is converted to a voltage by an impedance amplifier (TIA), which also provides a virtual ground for the column wires [108]. **c** The original image used for compression was input into the crossbar for two-dimensional (2D) DCT block by block. The white arrow shows the block processing sequence. The lower image shows a representative image block to be processed [108]. **d** Left: Image blocks were converted to voltages applied to the row lines of the crossbar, with neighboring lines having voltage pairs of the same amplitude, representing image pixel intensities, but opposite polarity. Right: Differential DCT wrote to a 128 × 64 array, with a small number of stuck "on" or "off" memristors visibly disrupted by the pattern [108]. **e** Images decoded from 2D DCT by software and **f** experiment. Before decoding, only the frequencies representing the top 15% of the spectral intensity (20:3 compression ratio) were retained [108]. **g** (i) Reference image. (ii) Images obtained using the direct mapping in [108–110]. (iii) Images obtained using the proposed 2D DCT reconstruction [111]. **h** Schematic illustration of a proposed physical crossbar array implementation and read circuitry [112]. **i** From top to bottom are the initial 50 k-byte image as the simulated input, the intermediate image representing the fuzzy logic level processing and the final 25 k-byte image after mapping back to the binary bitmap [112]

Image compression is an important image processing technique that eliminates redundant data and converts captured data into a manageable size for efficient processing and transmission. Image compression is achieved by reducing spatial redundancy, spectral redundancy, and temporal redundancy between data, and this process requires many matrix operations. Discrete wavelet transform (DWT) and discrete cosine transform (DCT) are widely used for image compression applications. Image compression using DCT is based on the principle of retaining the low-frequency signals which represent the main information content and removing the high-frequency signals denoting the image details and edge information. Hong et al. [107] proposed a one-dimensional DCT (1D-DCT) and one-dimensional IDCT (1D-IDCT) computational circuit from the angle of analog circuitry. The 2D circuit was designed based on the 1D circuit using parallel processing (Fig. 5a) and analyzed by simulating the circuit, which has an average computational accuracy of over 98.4%, and the average computation time has been reduced from milliseconds in MATLAB to subtle level. The proposed 2D circuit was applied for image compression. Considering that the complexity of DCT transform is relatively high in actual image compression, the common practice is to divide the image into multiple blocks, then perform DCT transform and inverse transform on the images in each block, followed by combining these blocks to improve the efficiency of the transform. Considering that larger blocks could reduce the effect of image blocks, 8 × 8 blocks are mostly used. It was found that the compression effect was significant at high speed.

DCT has a superior performance in terms of energy compression; but the entire calculation process is more complicated, which increases the burden on the calculation process. Compared with DCT, DWT exhibits a higher peak signal-to-noise ratio (PSNR) and faster image compression speed. However, traditional image compression methods, such as JPEG2000, require complex hardware to implement the calculation process. Therefore, directions such as reducing computing energy, required area, and image quality have become research hotspots.

Li et al. proposed a large-scale memristor crossbar switch for analog computing [108, 110] to achieve image compression by structuring an array of memristors up to 128 × 64 crossed hafnium oxide (HFO_2_) memristors [59] with sufficient accuracy and high-speed energy efficiency to realize analog vector multiplication. The proposed memristor array structure is presented in Fig. 5b, where the researchers construct a "1T1R" model, i.e., a memristor is integrated into a single piece on top of a metal oxide semiconductor transistor as an access device in each cell, for precisely adjusting the conductance of each memristor in the crossbar. The original compressed image was input into the array for pre-processing (Fig. 5c), the voltage was corresponding to the conductance value of the memristor, and vector matrix multiplication was performed (Fig. 5d), comparing the compression effect of software and hardware, as shown in Fig. 5e, f. The advantage of this framework is that the memristor longitudinal hardware VMM can directly process the analog signal acquired from the sensor, without the additional peripherals such as analog-to-digital converters (ADCs) and consuming additional time and energy. In addition, it can provide threshold gating circuits at considerably lower latency and energy cost, if only specific features need to be detected in the signal. This flexibility, along with low latency and high energy efficiency, makes analog longitudinal computing ideal for diverse edge and IoT computing.

To overcome the drawback that series computations are vulnerable to errors, Zhang et al. [111] fundamentally rethought how to implement image compression using resistive cross arrays (RCAs). The key idea is to reorganize the computation so that it natively matches the characteristics of the underlying resistive hardware, while the employed spectral optimization technique, quantization optimization technique, and 2D DCT reconstruction technique improve the robustness to errors for high-speed and efficient small-module processing. Meanwhile, simulation results showed that the quality of image processing was significantly improved (Fig. 5g), while the latency and power were reduced by 21% and 62%, respectively, facilitating the large-scale utilization of RCA with cost reduction requirements.

We compared multiple image compression techniques by two different datasets (Berkeley segmentation dataset and standard dataset) in terms of compressed quality parameters (MSE, PSNR, and SSIM), compression ratio, latency, power, and area as shown in Table 3. Here, D stands for direct mapping [108, 110]. D-P stands for the D method but its implementation is pipelined to maximize the throughput [109]. R stands for the proposed framework which is applied only with 2D DCT reconstruction. RF stands for the R method extended by spectral optimization. RFQ method is the RF method extended by hardware-friendly quantization. The normalized performance is shown in bold in the table, and according to Eqs. (5), (6), (7), the high image quality is featured by lower MSE, higher PSNR and SSIM. Tabulated results show that the proposed model has an image quality very similar to that of the human eye despite a slightly higher MSE and slightly lower PSNR and SSIM. Compared to previous work in [108, 110], the image quality is improved while the latency and power consumption are reduced by 51% and 24% or 3% and 61%, respectively.

**Table 3:** Comparison of different image compression techniques [111]

Name	Method	Image quality			Compression (BPP)	Latency (ms)	Power (mW)	Area (mm^2^)
		MSE	PSNR	SSIM				
Berkeley segmentation	Ideal	73.7	30.3	0.930	3.6	–	–	–
	D	223.5	25.1	0.757	6.3	0.61	140.8	0.077
	D–P	223.5	25.1	0.757	6.3	0.31	281.6	0.154
	R	89.1	29.4	0.908	3.2	0.25	140.8	0.077
	RF	84.8	29.6	0.913	3.3	0.25	121.8	0.063
	RFQ	83.2	29.7	0.916	3.3	0.25	107.9	0.063
Norm	Ideal	0.88	1.02	1.01	1.08	–	–	–
	D	4.29	0.79	0.75	2.17	2.06	1.30	1.23
	D–P	4.29	0.79	0.75	2.17	1.03	2.61	2.45
	R	1.08	0.99	0.99	0.97	**1.00**	1.30	1.23
	RF	1.03	0.99	**1.00**	0.99	**1.00**	1.13	**1.00**
	RFQ	**1.00**	**1.00**	**1.00**	**1.00**	**1.00**	**1.00**	**1.00**

Currently, integrated circuits that perform mathematical operations in artificial visual perception and image processing are mainly constructed of traditional digital logic gates. However, Boolean logic operations are not the most optimal alternative for brain computing, given the ambiguity possessed by biological neural networks. Previous studies have synthesized the optoelectronic properties of memristors and used a single optical gated memristor to build logic gates to realize logical OR and logical AND operations, while the important part of the logical NOT operation is missing in these gates, which requires a complicated operation to perform. Dan Berco et al. proposed a programmable photo-memristor gate [112], and this device can be used for image compression immediately during image acquisition, no additional memory modules are required (Fig. 5h, i). This design significantly reduces the number of processors and memories and time interactions. The smallest module of the designed structure consisted of two memristors and a resistor, which were used as building blocks in the design and simulation of the matrix multiplication unit by using logical operations (NIMPLY-AND) to form an effective in-situ compression of the image. However, the framework only performed single-channel image processing, and its effectiveness for more complex image processing was not explicit. The photoelectric properties exhibited by memristors in this case provide a new way of thinking for the development of intelligent vision.

### Image segmentation

Image segmentation, the division of target regions in an image from other regions as the name implies, is a crucial pre-processing for image recognition and computer vision. Among them, edge detection has become an overwhelming approach to image segmentation due to its distinguishing feature of different gray levels at the boundaries. Edge information is frequently utilized in image analysis, recognition and understanding. Therefore, edge detection and extraction are particularly important in image processing, and this technique is common in medical imaging, face and fingerprint recognition, traffic control systems and so on.

**Fig. 6:**
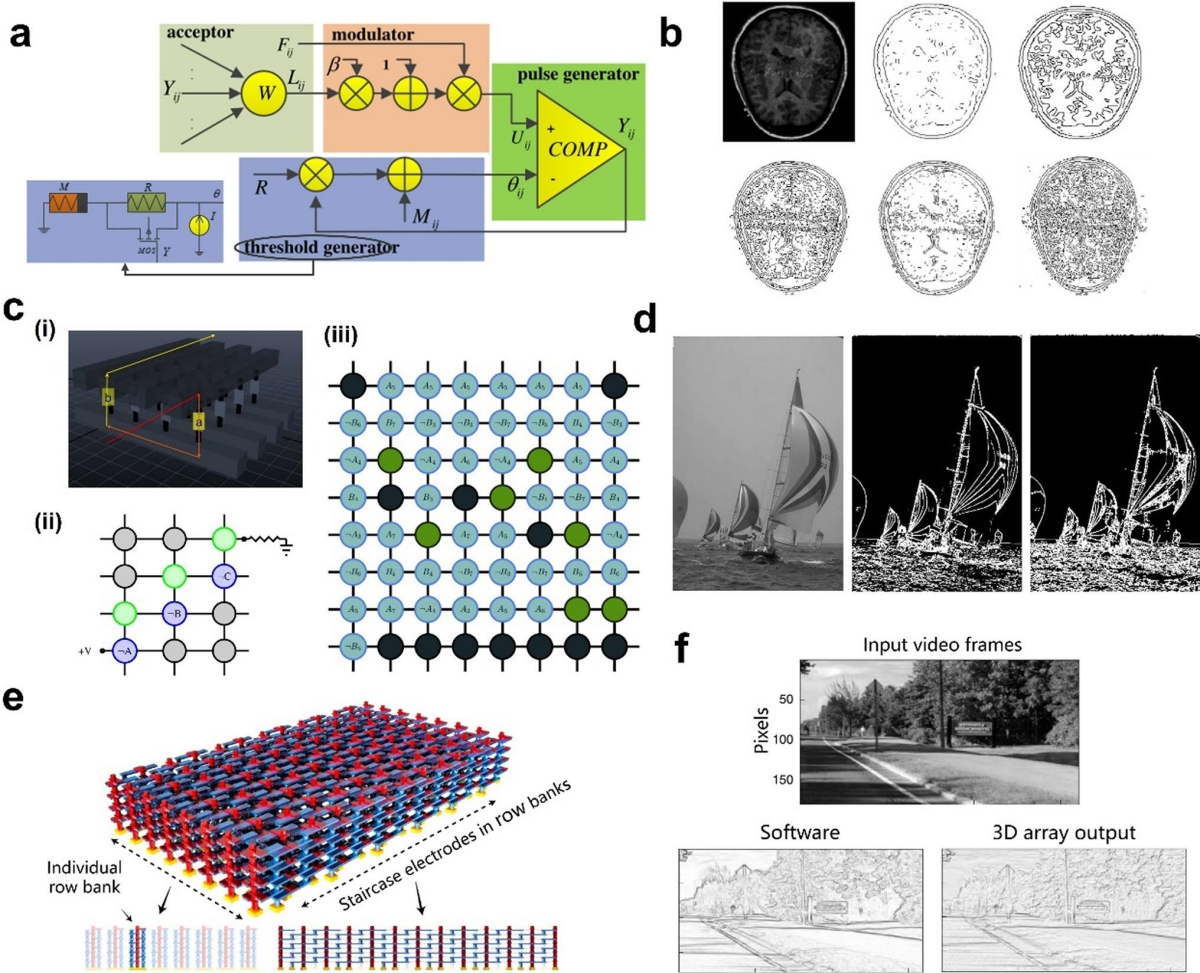
**a** Circuit diagram of the proposed M-PCNN structure and the memorized threshold generator circuit [34]. **b** Extraction of edges of CT images using different methods, in order from top to bottom from left to right: source image, using LOG operator, using canny operator and using the proposed M-PCNN with different memorizer parameters [34]. **c** (i) Flow-based computing with crossbar circuits. (ii) Crossbar design. (iii) Crossbars for edge detection on input-aware pixel pairs (median PSNR = 6 dB) [113]. **d** The input grayscale image, the computed edge image and the output edge image obtained via majority-based combination of approximately correct input-aware crossbar outputs, respectively [113]. **e** Schematic of the 3D circuits composed of high-density staircase output electrodes (blue) and pillar input electrodes (red). Sideview of 3D row banks and column side showing unique staircase electrodes. Each row bank in the 3D array operates independently [36]. **f** Comparison between the hardware and software edge detection of video frames [36]

Image edge detection extraction contributes to clinical diagnosis, and to address the shortcomings of traditional medical image fusion algorithms, Zhu et al. [34] constructed a memristive pulse-coupled neural network (M-PCNN) for medical image processing, and memorized threshold generator circuit is shown in Fig. 6a. The principle is that when an image is input to the M-PCNN, spiking neurons transmit stimuli to neighboring neurons and impel them to release pulses [114] to detect grayscale mutations of edges, which in turn enables edge detection. The edges were found clearer and richer obtained by using M-PCNN in medical image edge extraction (Fig. 6b). In addition, the integration of memristors into PCNNs significantly reduces the size of PCNNs while making the network biologically functional, which may facilitate the development of hardware implementations of neural networks. Although the core of the architecture was M-PCNN, which could simultaneously exploit the properties of specific linear additivity and nonlinear multiplicative coupling, allowing the introduction of a memristor to bring the network closer to a biological neural network, the peripheral circuits are needed to be redesigned for achieving the results of the different image processing, and the impact of the peripheral circuits on each processing method could not be ignored.

Chakraborty, D.'s team [113] explored the design of a stream-based cross-circuit with approximately correct input perception, producing multiple 8 × 8 cross-switching circuits (Fig. 6c-iii) belonging to two groups, one of which performs approximate edge detection for a specific application subset of the input values, and the other executes threshold-based edge detection for all possible pixel pairs with a high degree of accuracy (~ 85% accuracy). Outputs from the individual crossbars are combined using the majority function to yield the final output image. Figure 6c-i depicts a flow-based computation using the simple Boolean formula "a AND b" [115], where the data are added to the two-dimensional array of nanoscale memristors (Boolean operation is performed by adding the data to Fig. 6c-i a and b), and the current passing through the crossbar performs the desired computation. The current goes from the rightmost nanowire to the leftmost nanowire if and only if the formula "a AND b" is true. An example of a cross-switch that realizes the Boolean formula ¬A∧¬B∧¬C is shown in Fig. 6c-ii. Where the green circle indicates a memristor in the ON state, the gray circle indicates a memristor in the OFF state, and the blue circle assumes the value of literals. The team tested the edge extraction performance of the architecture on the BSD500 database [116] and utilized the PSNR metric to assess the quality of the output image, showing that the results (Fig. 6d) obtained from the input-aware approximation computation were significantly better than those produced by the more accurate general-purpose crossover. The cross array used for approximate computation, although effective in terms of accuracy and overall quality of edge extraction, adopts standard peripheral circuits and lacks exploration of the effect of peripheral circuits, which have an impact on the overall performance of the method in terms of efficiency and correctness.

Notably, most of the existing memristive systems are based on 2D arrays. As the units are only connected horizontally and vertically, such a 2D design sometimes cannot meet the complex topology of CNNs. Li et al. designed a 3D memristor circuit with a complex neural network shown in Fig. 6e [36], successfully extracted fine edge features using a 3D array and again obtained comparable results between software and hardware implementations of kernel operations (Fig. 6f). We found that despite the variability inherent in memristors, the actual processing results are comparable to software, while having pixel-level parallelism. The 3D array can be further expanded for parallel processing between different pixels, channels and filters over multiple convolutional layers. Compared to its 2D counterpart, this structure can conduct all computations in real-time and can be kernel vertically integrated directly to the 2D image sensor array, providing a significant speedup when running complex neural network models. This promises its application to cloud edge processors in IoT networks.

**Fig. 7:**
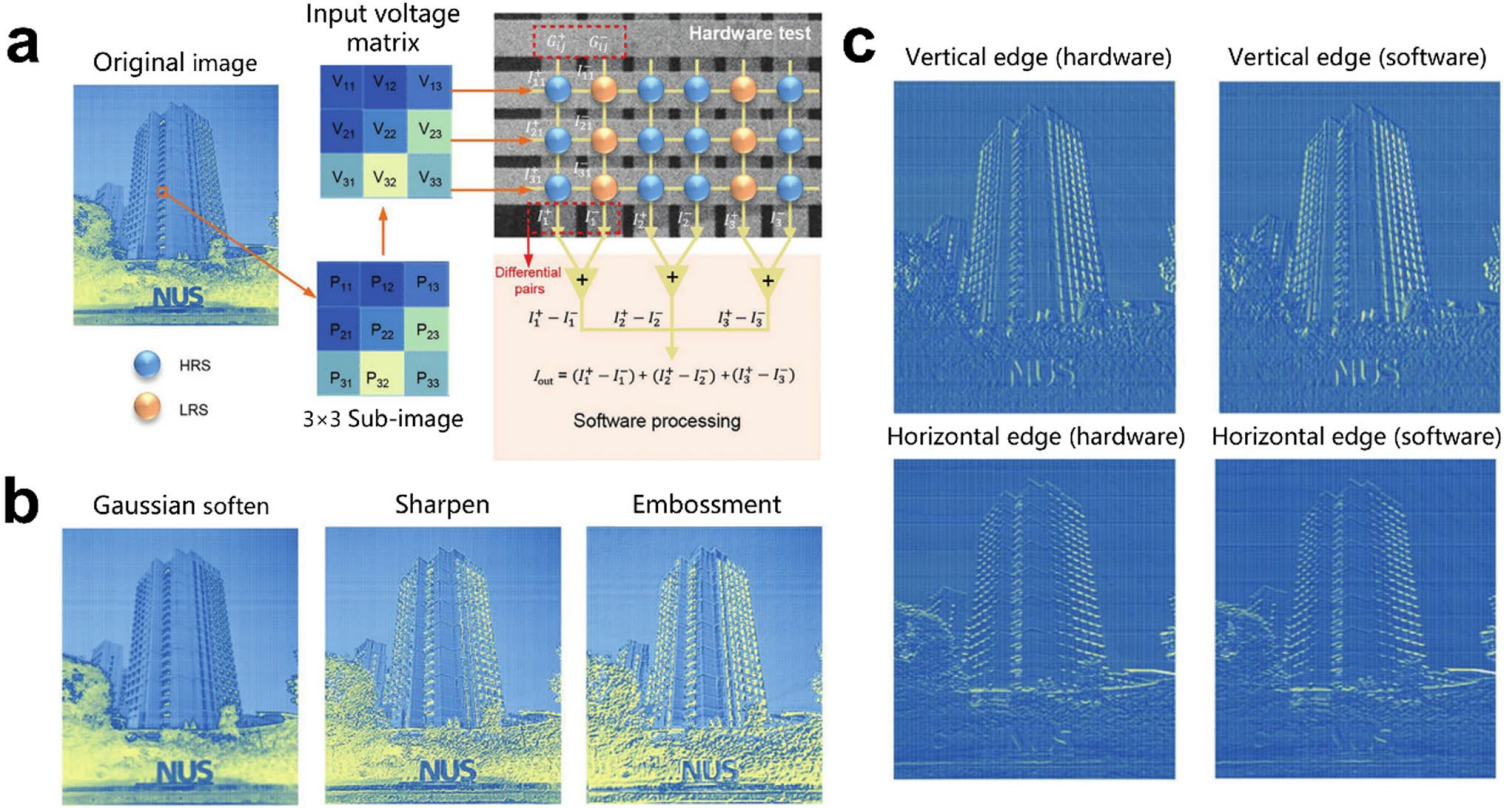
Convolutional image processing implemented using the PdSeOx/PdSe2 memristor crossbar array. **a** The whole process of convolution image processing using the memristor longitudinal array. **b** Results of image processing in five states implemented by adjusting the compliance current, mapping the weights of [-4 to 4]. **c** Hardware and software processed vertical and horizontal edge extractions. The Prewitt kennels are for horizontal and vertical edge detections [37]

The randomness of ion transport in traditional oxide-based memristors introduces variability to the system, which makes it challenging for CNNs to operate in memristor arrays, affecting the learning accuracy. To overcome this challenge, researchers have developed new structural memristors such as 2D and 3D. Li et al. proposed a 2D heterostructure memristor array [37] due to the unique physical properties of 2D materials, 2D material memristors exhibit better scalability. The team confirmed that the nine memristors in the 3 × 3 cross-array (Fig. 7a) were able to achieve a uniform and consistent five-state map by adjusting the compliance current. The intensity of the original image was converted to voltage and input to the memory array for convolution calculation to extract the edges of the image, as shown in Fig. 7c, where in the hardware processing results are similar to the software processing. Figure 7b also shows other image processing results, such as Gaussian softening, sharpening, and embossing. These demonstrate the potential of CNN operating in a diaphragm array.

### Image enhancement and restoration

#### Enhancement and fusion

Image enhancement processing is a major branch of digital image processing. Many images are usually captured with poor visual effects because of the environment and other conditions, which requires image enhancement techniques to improve human visual effects, for example, extracting characteristic parameters of target objects from digital images, highlighting certain features of target objects in images, etc., which are beneficial to the tracking, recognition and understanding of targets in images. Gradually, image enhancement technology has been involved in various aspects of human life and social production, such as the aerospace field, biomedical field, industrial production, public safety field and so on. To obtain good performance, some traditional image enhancement algorithms, such as the de-hashing algorithms of Tan and Oakley [117], Tan [118], He et al. [119], Tarel et al. [120], Nishino et al. [121], Meng et al. [122], and Sulami et al. [123], must pay the cost of a relatively heavy computational burden.

**Fig. 8:**
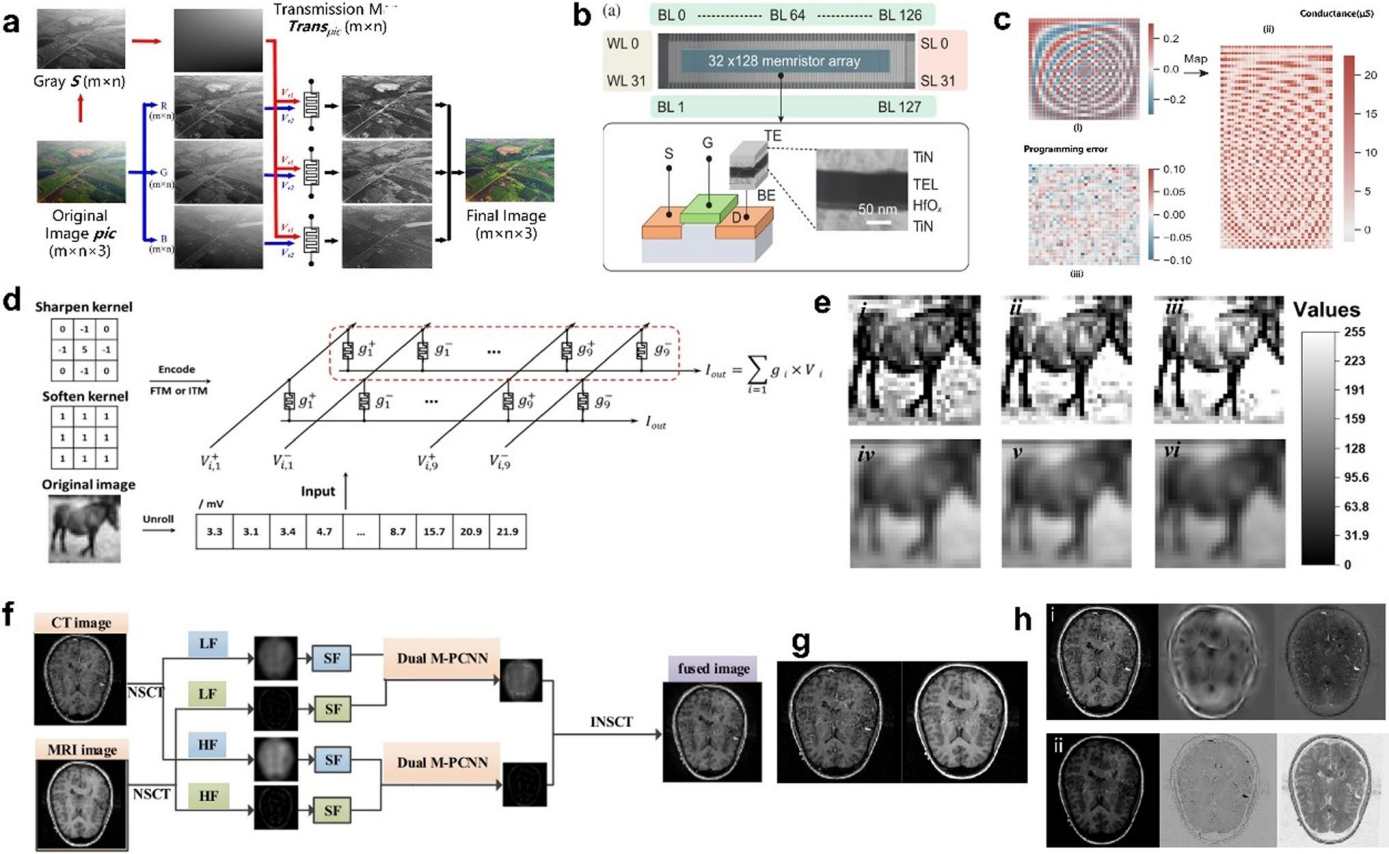
**a** MIEA overview: Red route: fine-tuning of the memristor crossover array using a rough image; Blue route: second fine-tuning of the architecture based on the original image. The final image was derived from current normalization [31]. **b** Structure of the device and 32 × 128 fabricated memristor array [32]. **c** Memristive array hardware system applied to image processing. (i) The origin DCT matrix; (ii) Array read current after processing by origin DCT matrix; (iii) the programming error matrix of (ii) [32]. **d** Specific image processing flow with Ag_2_S memristor arrays: encoding 3 × 3 convolution kernel values as array inputs, recording post-synaptic currents from the bottom electrodes after multiply-accumulate computation (MAC) operation as outputs, and mapping them to image grayscale values [33]. **e** Group 1: Sharpening operation. (i) Result of software-based simulation, hardware outputs of filament-type memristor (FTM)(ii) and interface-type memristor (ITM)(iii). Group 2: Softening operation: (iv) Software simulation result, hardware outcomes of FTM (v) and ITM (vi) [33]. **f** The fusing structure of NSCT-based M-PCNN [34]. **g** Source image: left: CT image, right: MRT image [34]. **h** Comparison of M-PCNN (i) and PCNN (ii) fusion results. From left to right are the fused images, the difference between the fused image and the CT image, and the difference between the fused image and the MRT image [34]

To avoid the maximum possible complex calculations generated by image enhancement, Zhu et al. [31] introduced memristor arrays into the image enhancement algorithm to subtly process images twice by the inherent properties of memristors. The algorithm uses a coarse transmission map and nonlinear memristor property with high efficiency, greatly reducing the computational cost, and the image quality evaluation reveals that it maintains a comparable performance with the classical algorithm (Fig. 8a). In addition, it was found that memristor-based image enhancement (MIEA) is more efficient than the classical algorithm in computing complexity and fine transmission map speeds. It takes 0.047 s on an Intel i7-9700 K CPU (14 nm), which is 90% less execution time than the 0.542 s in [119]. In the article [39, 48], the computational energy efficiency exceeds that of today's graphics processing units by two magnitudes. The presented processing fully exploits the memristor feature, but essentially the whole framework remains algorithmic. It is not considered to be a complete hardware-based digital image processing because the image pre-processing using memristors is only one step in the whole structure.

Later, Zhang et al. [32] proposed an array-level enhancement method that uses flexible combinations of multiple arrays to handle different layers of varying accuracy importance. 4096 1T1R cells, arranged as 32 × 128, were fabricated, as shown in Fig. 8b. This memristor array demonstrates the multi-level characteristics of the measurement. The size of the discrete cosine variation matrix was matched to the size of the memristor array, each element in the matrix mapped to the array, the voltages were input to the rows and the output results were generated by accumulating the currents in each row. Comparing the original discrete matrix with the current matrix produces the programming error (Fig. 8c). They stored the transformation matrix in the array and the performance of the image processing is sensitive to changes, which suggests that the array-level enhancement method can reduce the programmed multi-level data changes.

Zhu et al. [33] applied Ag_2_S flexible memristors to digital image sharpening and blurring, subtly adopting the switching mechanism of the device's different interface resistors. The processing principle was that original image pixel values (from 0 to 255) were linearly mapped to read voltages (amplitude from 0 to 25.5 mV), two convolution kernel values were mapped into a cross-array (Fig. 8d) to modulate the conductance value of the device, and the structure shared a common bottom electrode so that two types of currents could be collected—the current generated by current summation and the output current generated by the multiplication of voltage and conductance. The grayscale image of the final processed result (Fig. 8e software (i and iv) and hardware processing results (ii, iii, v, and vi)) was generated by output currents. Apparently, after the sharpening operation, the outline of the "horse" was clearer, while the softening operation blurred the outline of the "horse" and its surroundings. However, this method inevitably applies convolutional operations in the processing, which increases the computational complexity and requires multiple iterations of training, consuming more space in the case of larger convolutional kernels.

Image fusion, another level of image enhancement, refers to the extraction of the image data about the same target acquired by multiple source channels through image processing and computer technology, and so forth, to maximize the favorable information in the respective channels and finally synthesize them into a high-quality image, reducing the uncertainty and redundancy of the output. The research of image fusion technology is on the rise, and the application fields are spread over remote-sensing image processing, visible light image processing, medical image processing, infrared image processing, and so on. Zhu et al. [34] fused CT and MRI images by using M-PCNN, whose structure is shown in Fig. 8f. The fusion process consists of the following four steps: first, to avoid the blending phenomenon of low-frequency subbands and thus overcome the pseudo-Gibbs phenomenon [124], the CT images and MRI were sampled by using the non-sampled contour transform (NSCT); second, stimulating M-PCNN neurons by utilizing the spatial frequency of NSCT transform domain coefficients. Again, the coefficients, with a high ignition number as the coefficients of the fused images. Finally, the new images were fused using the inverse NSCT algorithm after fusing the high and low frequency coefficients of the two images by the two-channel M-PCNN. The results of image fusion of the original image (Fig. 8g) using PCNN and M-PCNN are shown in Fig. 8h. The subjective visual comparison shows that M-PCNN for medical image fusion has a superior fusion performance, from which more detailed information can also be obtained.

#### Restoration

Similar to image enhancement, the restoration of images also targets at improving the overall quality of the image. Instead of image enhancement techniques, which focus on increasing the contrast and processing the image according to the receiver's preference, image restoration focus on removing the blurred parts of the image and repairing or reconstructing the degraded image, which can be considered as the reverse process of image degradation.

**Fig. 9:**
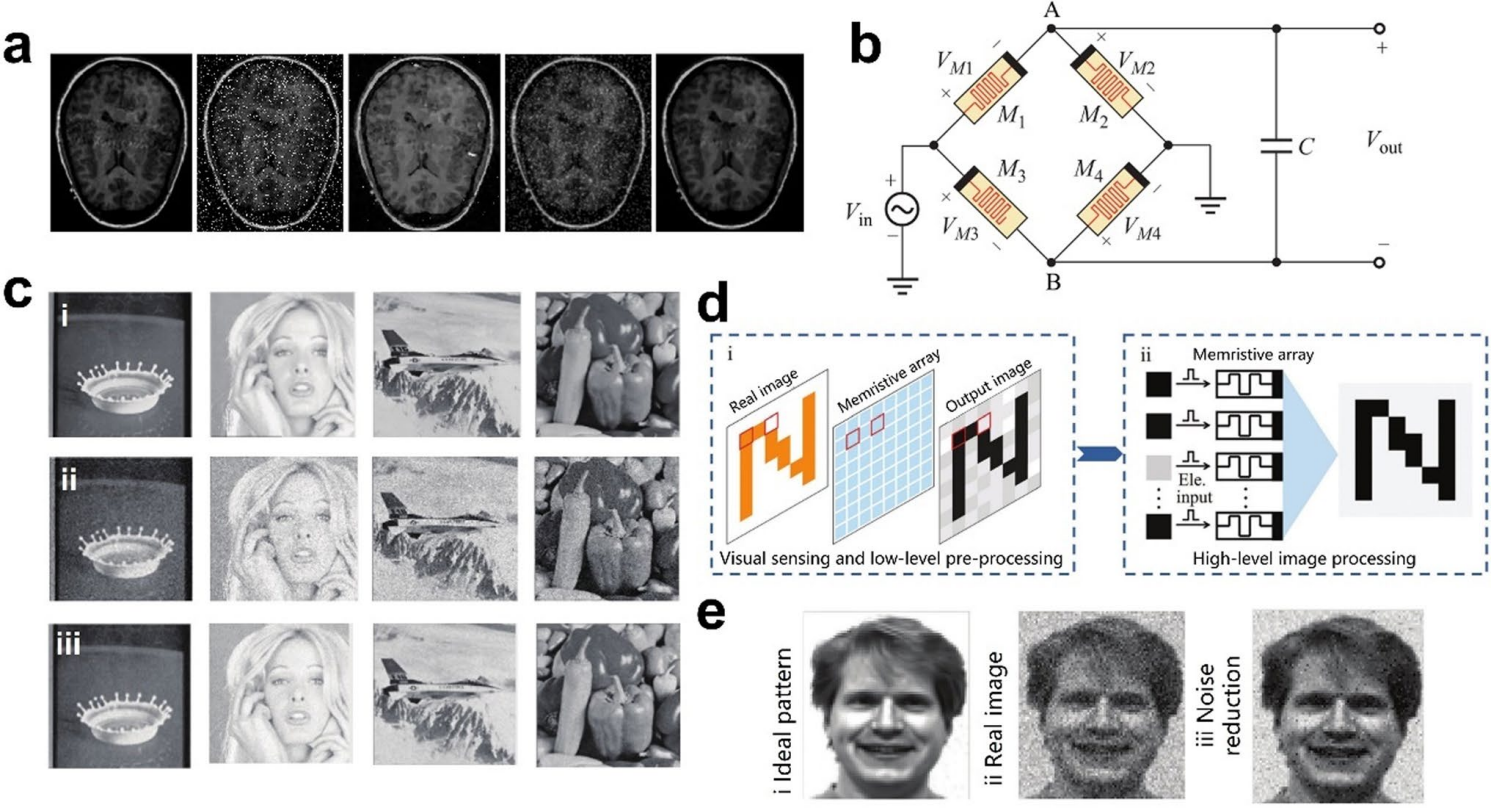
**a** M-PCNN for medical image denoising. In order, the source CT images, images with salt and pepper noise, images processed by median filter denoising, images denoised by averaging filtering, and images denoised by M-PCNN [34]. **b** New LPF based on memristor bridge [126]. **c** Testing images and filter results. i) Standard clean images. ii) Image with white Gaussian noise. iii) Denoise by proposed adaptive Gaussian filter [126]. **d** Illustration of a neuromorphic visual system utilizing plasma photoelectric memristors for visual sensing, low-level image pre-processing, and high-level image processing (i.e., recognition) [35]. **e** Images comprising the ideal image (i), the real image with 10% random noise (ii) and the noise-reduced image (iii) after pre-processing. Image taken from the Yale Face Database B [127] [35]

Considering that the noise generated during medical imaging degrades the image quality and blurs the observed tissue boundaries, which affects medical diagnosis, it is important to remove the noise while preserving the boundary and structural information. Zhu et al. [34] used the proposed M-PCNN structure for image denoising, where each neuron is connected to the corresponding pixel point and is also to the adjacent 3 × 3 neurons [125]. In most cases, the correlation between pixel values of noise and surrounding is weak and significantly different, and neurons mainly have two states: stimulated and unstimulated. The main principle of image denoising using M-PCNN is to judge and distinguish the noise, i.e., to judge whether each neuron and its neighboring neurons are stimulated or not. The obtained result adjusts the brightness of the corresponding pixel values for the purpose of noise reduction and image recovery. M-PCNN was proved to be a more suitable method for removing pretzel noise and retaining edge information, as seen through comparison (Fig. 9a).

Suppressing the high-frequency signal while passing the low-frequency signal is the main function of the low-pass filter (LPF), an important part of image denoising [128]. Yongbin et al. [126] designed a new LPF based on memristor bridge circuit described in [129], consisting of four identical memristors that can perform zero, positive and negative synaptic weightings (Fig. 9b). They discussed the memristor bridge-based LPF with its cutoff frequency varying over time and found a way to design a memristive Gaussian filter and its application to image processing inspired by the memristive filter and its typical characteristic. The principle is that the adaptive Gaussian kernel will change in different situations and the character of their LPF with variable cutoff frequency, which can be combined with the Gaussian filter to denoise the image. The researcher added Gaussian white noise with standard deviation σ = 10/255 to the 512 × 512-pixel image, and the Gaussian template size was set to 11 × 11. Then, the Gaussian filter proposed in the literature [130] and designed adaptive Gaussian filter were respectively used to denoise these noisy images, and the filtering results are shown in Fig. 9c. The designed filter circuit combined the variable parameters based on the memristor bridge with Gaussian filters, which provided a new idea for the image-filtering algorithm. However, the problem of the memristor being only a piece of the puzzle rather than the overall system architecture remains, and the purpose of hardware implementation is not completely reached.

Recently, the authors developed a plasmonic photomemory in Ag-TiO_2_ nanocomposite films that relies on optical excitation and the effects of localized surface plasmon resonance [35]. Such a device can integrate visual perception, low-level image pre-processing (including noise reduction and contrast enhancement), and high-level image processing functions (Fig. 9d). (Fig. 9d). They utilized an 80 × 80 phototransistor array to construct a neuromorphic vision system that comprised of two components. The preprocessed images were fed into an artificial neural network with two layers of nerves based on photomemristors to implement image learning and recognition (Fig. 9d-ii), and the low-level preprocessed images were obtained (Fig. 9e). The images show that the background noise was further smoothed after noise reduction.

### Image recognition and classification

Image recognition, one of the mechanisms of computer vision, is based on the main features of an image and is a technique that analyzes the original image overall to reach a prediction of the category that it belongs to. In human image recognition, it is necessary to exclude redundant information from the input, extract the key information and integrate the information obtained at each stage to obtain a complete impression. The image recognition process is similar to it. One of the most important models for image recognition is the convolutional neural networks, but it has not yet been fully hardwared through memristor crossbars [131], an array of crossbars with memristor devices at each intersection. In addition, it is extremely challenging to achieve software-equivalent results because of its high variability, low device throughput, and other non-ideal characteristics [132–135].

**Fig. 10:**
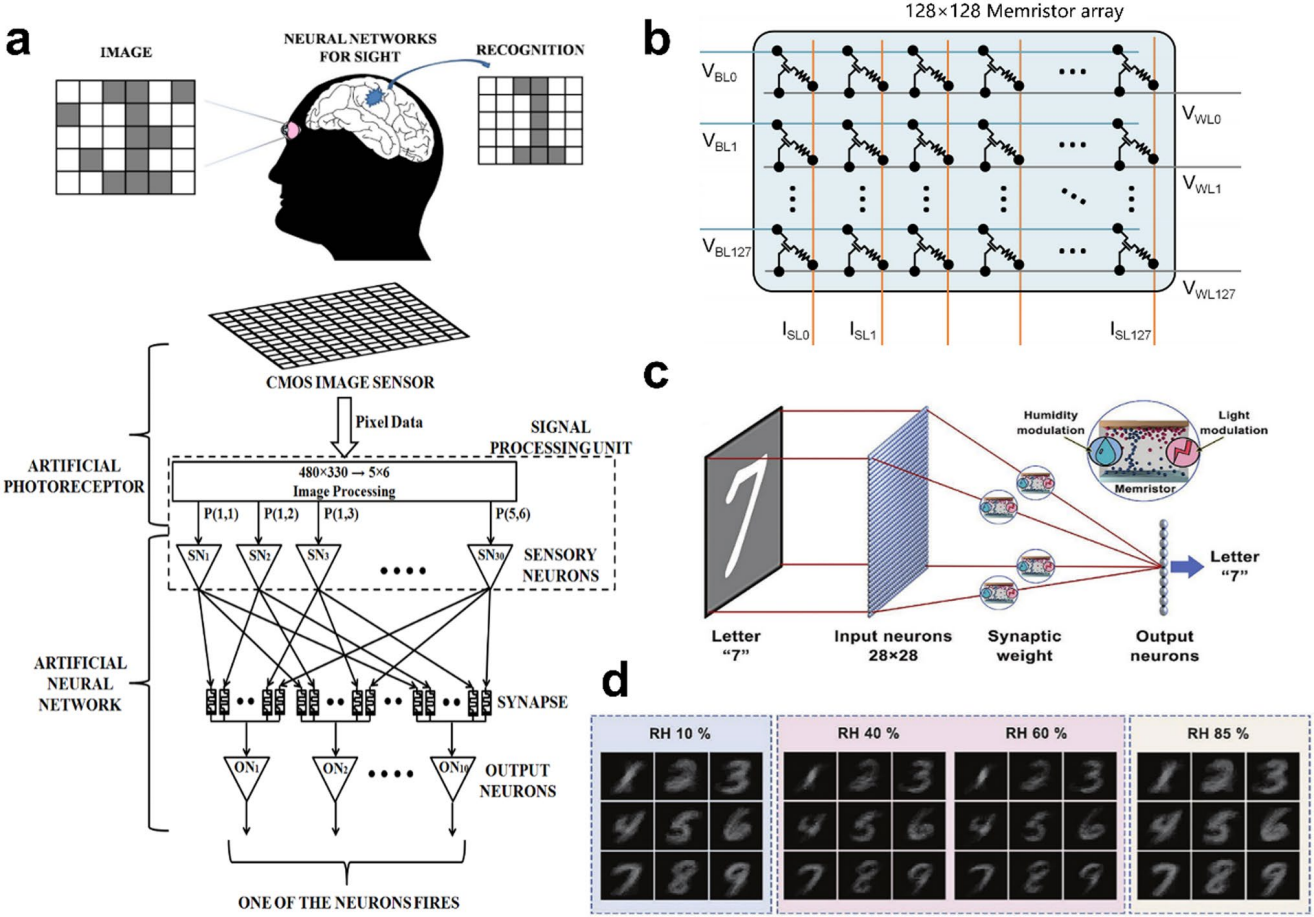
**a** Neuromorphic system for visual pattern recognition [38]. **b** Architecture of the simulated memristor-based neural processing unit [39]. **c** Schematic illustration of the high-level in-sensor computing by employing the sensing memristor as a synapse to implement weight updating [40]. **d** Output image after 60,000 training epochs for the sensing memristor under different RH levels [40]

Chu et al. [38] designed a visual pattern recognition neuromorphic hardware system (Fig. 10a), which consists of an artificial photoreceptor, a PCMO-based memristor array and CMOS neurons. Among them, an artificial photoreceptor converted images into input voltage pulses, memristor arrays [136] were used for synaptic connections, and leaky integrate-and-fire (I&F) neurons serve as output neurons. An improved spike-timing dependent plasticity algorithm was proposed for accordingly adjusting the memristor states or synaptic weights during system training. The system operated on the principle that when one of the output neurons integrated the current flowing through the memristor, it would reach a certain threshold earlier than the other neurons. An inhibitory signal from the discharged neuron would freeze all neurons and reset their internal state so that the recognition process could be restarted with the next test image. The system has been successfully trained and recognized digital images from 0 ~ 9, and random noise was added before applying the training images to the system for recognition, after which it was found that as the noise level increases, the recognition rate decreases correspondingly, for example, to 85% at 10% noise level. Adjusting the resistive state or synaptic weights of the memristors with algorithms during the training of the system, however, invariably increases the training time and cost. More attention needs to be paid to this point in the future implementation of memristor-based systems for hardware digital image processing as well.

Then, Yao et al. [39] fabricated a memristor cross-array implementing CNNs that integrated eight 2048 one-transistor-one-memristor (1T1R) arrays and constructed a complete memristor-based five-layer CNN for MNIST image recognition [137], with an experimental correct recognition rate of 96.19% using this hybrid training scheme on the entire test dataset. In addition, the convolution kernel was replicated to three parallel memristor convolvers to reduce the mCNN latency by roughly one-third. In addition, the memristor-based CNN neuromorphic system (Fig. 10b) was shown to be is more energy efficient than state-of-the-art graphics processing units by over two orders of magnitude, and its highly integrated neuromorphic system provides a feasible solution. While improving CNN computational efficiency, it is expected to provide a plausible non-Von Neumann hardware solution for edge computing and deep neural networks, as well as being memristor-based. Given that present research on the neuromorphological aspects of memristors has mainly focused on single sensory processing such as vision, hearing, smell, and touch, whereas the human perceptual system can perceive and process diverse types of information simultaneously in complex environments at the same time. Wang et al. proposed an MXene-ZnO-based multimodal flexible sensing memristor that combines visual data sensing, relative humidity (RH) sensing, and pre-processing functions [40]. In the simulation, single-layer perception (SLP) consists of 28 × 28 neurons (785 input neurons), ten output neurons (ten categories, from 0 to 9), and fully connected 785 × 10 synaptic weights. Presynaptic neurons perceived inputs of 28 × 28 MNIST digits and transmitted them as synaptic forward potentials. The synaptic weights were modulated by humidity modulation and light modulation, to which the post-synaptic neurons then responded and performed the perceptual task (Fig. 10c). The researchers found that the artificial vision network trained at 60% RH captured more features from selected alphabets compared to low/high humidity (Fig. 10d) and could achieve high recognition accuracy after 60,000 training cycles. These results show that multimodal sensing memristors can be applied to both low and high-level sensor computing by reducing power consumption and chip area.

## Discussion

Memristor, a new type of non-volatile storage element, is expected to constitute a new type of image processor to accelerate the process of image processing. Applying the memristor network to image processing has the following advantages: from the device level, firstly, the memristor, with its non-volatility (the stored information is still maintained after the power is cut-off), can be used to store the model parameters and the intermediate results, which can be applied to realize a more efficient data transfer and persistence in image processing tasks. For image processing tasks that require real-time performance, such as real-time target detection and tracking, memristors possess very fast response speeds, and therefore memristor networks can realize high-speed image processing and analysis. In addition, because of their particular properties, memristor networks can better mimic the synaptic connections in the biological nervous system, which can be used in image processing to achieve a process that is more similar to that of the human visual system. On an architectural level, for image processing in mobile devices and embedded systems, the low energy consumption properties of memristors can contribute to prolonging the battery life of the devices. For robust image processing in noisy environments, the nonlinear characteristics of the memristor can be used for noise suppression and filtering, and it is tolerant to noise in the input data. Additionally, the structure and characteristics of the memristor networks can support massively parallel computation for processing tasks that require dealing with a large amount of data, thus accelerating the image processing task.

Despite the advantages of a system framework for image processing via memristor networks, there are still several challenges in implementing hardware applications of digital image processing in neuromorphic systems. Due to the nonlinear properties of the memristors, their incorporation into the Modified Nodal Analysis (MNA) structure results in the system of circuit equations becoming a nonlinear system of equations and increasing the complexity of the solution. Solving the nonlinear equation system usually requires the use of numerical computation methods, such as Newton's method and the Quasi-Newton Methods, which consume more computational resources and time as compared to the methods for solving the linear equation system. In addition, the memristor's memristor value may vary with time and operating state, which will increase the complexity of analyzing the dynamic behavior of the circuit. For the I–V characteristics of memristors, it may be necessary to use more precise and complicated models for modeling and matching, which also increases the challenge of circuit analysis. Therefore, it is necessary to comprehensively consider the computational resources and time, the accuracy and efficiency of circuit analysis when designing a large-scale memristor network.

In the former study, we found that in the hardware implementation of neural networks, the offline training approach requires the use of computer-assisted training of the neural network to obtain the weight update values, and then adjust the resistance values of the memristor arrays, and such frequent hardware and software data interactions do not provide real-time weight updates. However, as the scale of the number of cells in the neural network increases, for example, when a cellular neural network needs to be utilized to process a large-size image, the circuit structure of the neural network becomes complicated, which can cause inconvenience in updating the weight templates. Therefore, it would be more practical to transfer the training algorithm from software to hardware and to be able to update the resistance value of the memristor in real-time.

Meanwhile, how to fabricate networks on a large scale with memristor devices and how to determine the relationship between the size of that network and the actual image pixel size. The current use of memristor networks for slightly larger pixel digital image processing almost always slices the image, corresponding to memristor networks as small arrays, which will generate a new problem, no connection between individual blocks, meaning that the whole image cannot be processed. Thus, how to link the impact of a single pixel in a digital image to the surrounding pixels for better performance must also be addressed in subsequent studies of this system architecture. To this end, we can consider the interactions that exist between the memristors and apply them in digital image processing, which can reduce the network computational difficulty and the influence of peripheral circuits. When using the memristor system network to process pictures, how to effectively input the data into the array on a large scale and realize the integration and high efficiency of the whole memristor-based digital image processing network is also a problem that needs to be solved in the subsequent research. To tackle this challenge, the design of a batch write and read module is an optimal choice; however, the design simulation and debugging of the read and write module in SPICE is also a considerable amount of work. Currently, researchers have a long way to go in the field of memristor network-based digital image processing.

We are convinced that the memristor network for image processing capability would also be enhanced with the constant advancement of memristor technology and the future tendency of memristor networks in image processing may be the following. First, memristors could rapidly process and analyze large-scale image data because of their efficient data storage and processing capabilities in image processing, which may also be extensively applied in real-time image processing, such as video processing, machine vision, etc. Memristors feature low power consumption and high energy efficiency, which can be better integrated with other low-power image processing technologies, like deep learning, to achieve highly efficient and energy-saving performance. In addition, memristors can be incorporated with deep learning technologies and other technologies to realize more high-efficiency artificial intelligence algorithms and be broadly used in image recognition, autonomous driving, smart home and other domains. The study of the experimental and physical mechanism theory of memristors will lay a firm foundation for their application in the field of digital image processing. We believe that experimental optimization of the memristor fabrication process, combined with theoretical device simulation tools, could provide an operational non-Von Neumann hardware solution for hardware image processing and neuromorphic networks.

## Conclusion

The conventional von Neumann computer architecture has become gradually less efficient as the demand for high-performance computing in the domain of digital image processing grows. This paper, on the premise of reviewing the commonly employed classical algorithms for digital image processing, reviewed a novel hardware architecture of memristor-based networks that is different from the algorithms. Its principle is based on the fact that the properties of the memristor device itself simulate the mechanism of retinal operation in the human visual system, i.e., the augmentation and inhibition between neurons. Subsequently, we elaborated on the properties of the memristors used in digital image processing, and their possible physical behavioral mechanisms. In addition, we detailed the state-of-the-art applications of memristor networks in digital image processing, involving image logic operations, image compression, image segmentation, image enhancement and restoration, and image classification and recognition. Compared with conventional digital image processing architectures, networks based on memristors, a key component in artificial visual systems, have the advantages of lower power consumption, smaller size, higher integration, and better flexibility. In addition, image processing networks constructed by introducing memristors feature faster processing times, higher efficiency, and better reliability than pure software algorithm architectures alone. This review guides the study of the application of using memristors and their neuromorphic properties in hardware digital image processing.

## Data Availability

Data can be obtained from authors under reasonable request.
